# The Wall-Associated Receptor-Like Kinase TaWAK7D Is Required for Defense Responses to *Rhizoctonia cerealis* in Wheat

**DOI:** 10.3390/ijms22115629

**Published:** 2021-05-26

**Authors:** Haijun Qi, Xiuliang Zhu, Feilong Guo, Liangjie Lv, Zengyan Zhang

**Affiliations:** 1 The National Key Facility for Crop Gene Resources and Genetic Improvement, Institute of Crop Science, Chinese Academy of Agricultural Sciences, Beijing 100081, China; haijunqi@yeah.net (H.Q.); zhuxiuliang@caas.cn (X.Z.); guofeilong1117@163.com (F.G.); 2 Institute of Cereal and Oil Crops, Hebei Academy of Agriculture and Forestry Sciences, Shijiazhuang 050035, China; liangjie_lv@163.com

**Keywords:** defense response, *Rhizoctonia cerealis*, wall-associated receptor kinase, wheat (*Triticum aestivum*)

## Abstract

Sharp eyespot, caused by necrotrophic fungus *Rhizoctonia cerealis*, is a serious fungal disease in wheat (*Triticum aestivum*). Certain wall-associated receptor kinases (WAK) mediate resistance to diseases caused by biotrophic/hemibiotrophic pathogens in several plant species. Yet, none of wheat *WAK* genes with positive effect on the innate immune responses to *R. cerealis* has been reported. In this study, we identified a *WAK* gene *TaWAK7D*, located on chromosome 7D, and showed its positive regulatory role in the defense response to *R. cerealis* infection in wheat. RNA-seq and qRT-PCR analyses showed that *TaWAK7D* transcript abundance was elevated in wheat after *R. cerealis* inoculation and the induction in the stem was the highest among the tested organs. Additionally, *TaWAK7D* transcript levels were significantly elevated by pectin and chitin treatments. The knock-down of *TaWAK7D* transcript impaired resistance to *R. cerealis* and repressed the expression of five pathogenesis-related genes in wheat. The green fluorescent protein signal distribution assays indicated that TaWAK7D localized on the plasma membrane in wheat protoplasts. Thus, TaWAK7D, which is induced by *R. cerealis*, pectin and chitin stimuli, positively participates in defense responses to *R. cerealis* through modulating the expression of several pathogenesis-related genes in wheat.

## 1. Introduction

Wheat (*Triticum aestivum*) is one of the most important staple crops [1]. The wheat sharp eyespot disease, primarily caused by the necrotrophic fungus *Rhizoctonia cerealis*, is one destructive disease for wheat in many regions of the world [2,3]. In China, sharp eyespot disease has become an economically important wheat disease in the past two decades; 6.67–9.33 million hectares of wheat fields are affected by this disease per year [4,5]. Breeding of resistant wheat varieties is the most environmentally friendly and reliable approach to control sharp eyespot. The resistance in wheat accessions is partial and quantitative [6,7,8]. No effective QTL can be available to breed varieties using traditional methods. To improve wheat resistance by molecular methods, it is crucial to isolate key resistance genes. 

Wall-associated kinases (WAKs) and WAKs-like proteins (WAKLs) belong to a plant-specific subfamily of receptor-like kinase family. Their protein sequences all include an intracellular serine/threonine (Ser/Thr) protein kinase domain, a transmembrane region, 0–2 epidermal growth factor (EGF) domains, and an extracellular galacturonan-binding (GUB) domain that closely connects to the cell wall pectin [9,10,11]. Some WAKs have been implicated in resistance to bacterial and fungal diseases. In *Arabidopsis thaliana*, *WAKL22* confers the dominant resistance to *Fusarium* wilt disease [12]. The maize smut resistance gene *ZmqHSR1*, which encodes a non-RD (non arginine-aspartate) WAK protein named ZmWAK, was highly expressed in mesocotyls of the resistant maize varieties [13]. The maize northern corn leaf blight (NCLB) resistance gene *Htn1* encodes another non-RD WAK protein (ZmWAK-RLK1) [14]. ZmWAK-RLK1 can improve the resistance of corn plants by reducing the production of benzoxazines [15]. In rice, the *OsWAK1* gene was found to be significantly induced during incompatible interaction with the rice blast fungus *Magnaporthe oryzae*, and *OsWAK1* overexpression in rice enhanced the resistance against *M. oryzae* [16]. Delteil et al. [17] revealed that four OsWAK proteins acted as regulators in rice quantitative resistance against *M. oryzae*. Of them, *OsWAK14, OsWAK91* and *OsWAK92* positively regulated quantitative resistance to *M. oryzae* [18]. Overexpression of *OsWAK25* enhanced resistance to both the hemibiotrophic pathogens *Xanthomonas oryzae* and *M. oryzae*, but led to increased susceptibility to the necrotrophic pathogens *Rhizoctonia solani* and *Cochliobolus miyabean* through activating expression of pathogenesis-related genes *PR10*, *PAL2*, *PBZ1* and *NH1* in rice [19]. A recent cloned the rice *Xa4* locus, encoding a wall-associated kinase, confers durable resistance against *Xanthomonas oryzae* pv. *oryzae* through promoting cellulose synthesis, suppressing cell wall loosening and strengthening of the cell wall [20]. In wheat, the *Septoria tritici* blotch (STB) disease resistance gene *Stb6*, which located on chromosome 3AS, encodes a WAK-like protein, confers resistance against infection of *Zymoseptoria tritici* [21]. Additionally, *TaWAK6*, a WAK-encoding gene located on chromosome 5B, confers adult plant resistance to leaf rust (*Puccinia triticina*) in wheat, although *TaWAK6* overexpression did not affect seedling resistance [22]. Our laboratory identified a wheat WAK gene *TaWAK5* responding to *R. cerealis* infection, but its silencing by VIGS (virus-induced gene-silencing) did not impair the resistance of wheat [23].

In this study, we identified a pathogen-induced *WAK* gene from wheat chromosome 7D, designed as *TaWAK7D*, and investigated its defense role in wheat defense response to *R. cerealis*, as well as analyzed the protein subcellular localization. These results suggested that TaWAK7D positively participated in the defense against *R. cerealis* infection through activating the expression of several pathogenesis-related (*PR*) genes, including *β-1,3-Glucanase*, *Chitinase3, Chitinase4, PR1* and *PR17*. 

## 2. Results

### 2.1. TaWAK7D Transcript Abundance in Wheat Is Responsive to R. cerealis

**Figure 1:**
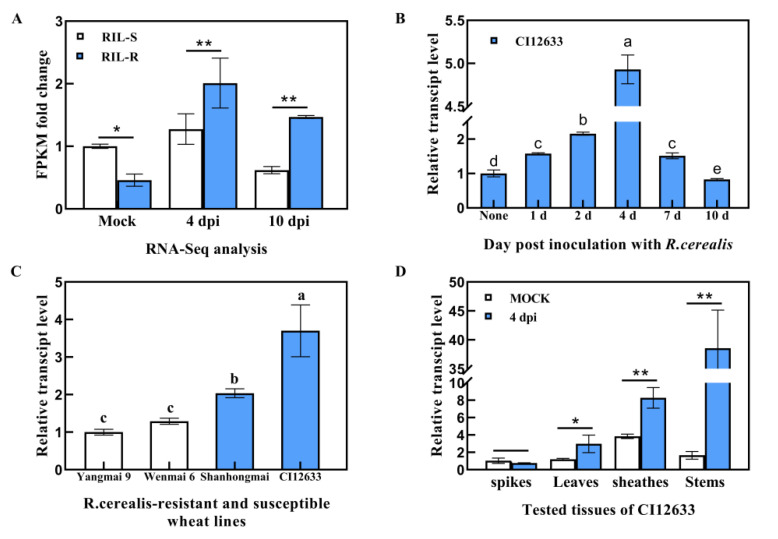
Transcript profiles of *TaWAK7D* responding to *Rhizoctonia cerealis* in wheat. (**A**) The transcriptional level of *TaWAK7D* was obviously increasing in the resistant lines (RIL-R) than in the susceptible lines (RIL-S) at 4 and 10 dpi with *R. cerealis*. The expression level of *TaWAK7D* in RIL-S plants at mock was set to 1. (**B**) Transcript profiles of *TaWAK7D* in *R. cerealis*-resistant wheat line CI12633 at none, 1, 2, 4, 7 and 10 dpi with *R. cerealis* Rc207. *TaWAK7D* transcript level of none was set to 1. (**C**) Expression patterns of *TaWAK7D* in four wheat cultivars with different resistance degrees at 4 dpi with *R. cerealis*. The expression level of *TaWAK7D* in Yangmai 9 was set to 1. (**D**) Expression pattern of *TaWAK7D* in spikes, leaves, sheathes and stems of CI12633 at 4 dpi with *R. cerealis* or mock. The transcriptional level of *TaWAK7D* in spikes with mock treatment was set to 1. *TaActin* was used as the internal control. Significant differences were determined based on three technical repeats (Student’s *t*-test: * *p* < 0.05; ** *p* < 0.01). Bars indicate the standard error of the mean.

By comparative analysis of RNA-seq data from resistant and susceptible lines of recombinant inbred lines (RILs) derived from the cross Shanhongmai × Wenmai 6, the gene with ID *TraesCS7D02G087000* was identified to be significantly up-regulated in the resistant RILs relative to the susceptible RILs after *R. cerealis* strain Rc207 inoculation. The gene transcript level were higher by 1.58- and 2.34-fold in the resistant RILs relative to the susceptible lines at 4- and 10-days post inoculation (dpi) with the fungus, respectively (Figure 1A). BlastP (https://blast.ncbi.nlm.nih.gov/Blast.cgi, accessed on 6 May 2020) and sequence analyses indicated that *TraesCS7D02G087000* was located on wheat chromosome 7D and encoded a wall-associated kinase. Hereafter, this gene was designated as *TaWAK7D*. 

qRT-PCR analysis showed that as shown in Figure 1B, after inoculation with *R. cerealis, TaWAK7D* transcript in *R. cerealis*-resistant wheat cultivar (cv.) CI12633 was significantly increased at 2 dpi and reached a peak at 4 dpi (~4.93-fold) compared with the untreated one, and then decreased to 0.83 fold at 10 dpi, implying that the gene may be involved in the early defense response to the fungal infection. Furthermore, *TaWAK7D* transcript level was the highest in the resistant wheat cv. CI12633, followed by the resistant wheat cv. Shanhongmai, and the lowest in the highly susceptible wheat cv. Yangmai 9 (Figure 1C). Additionally, tissue expression analysis showed that at 4 dpi with *R. cerealis*, the greatest induction appeared at the stems of CI12633 (Figure 1D), where sharp eyespot disease usually occurs. These results suggested that the *TaWAK7D* gene might participate in wheat defense responses to infection of *R. cerealis*. 

### 2.2. Sequence and Phylogenetic Analyses of TaWAK7D

**Figure 2:**
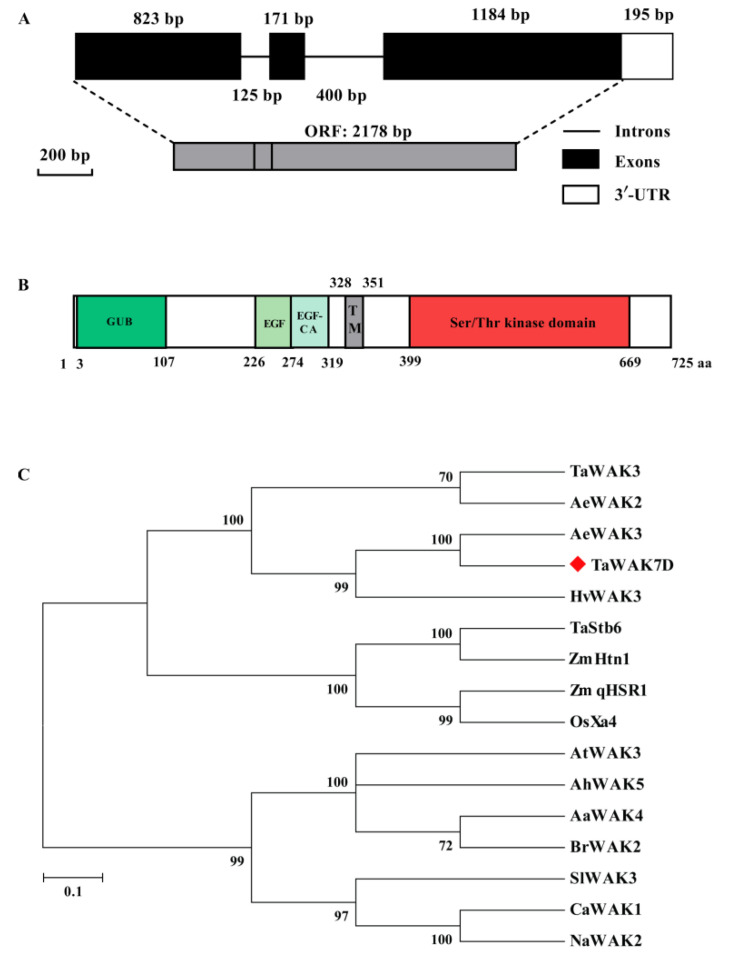
Sequence and phylogenetic analyses of TaWAK7D. (**A**) Gene structure of *TaWAK7D*. The white box indicates UTR, black boxes represent exons and black lines indicate introns. (**B**) Schematic diagram of the TaWAK7D protein. The colored regions indicate different domains. (**C**) A phylogenetic tree of TaWAK7D and 15 other WAK members from monocots and dicots. The bootstrapped phylogenetic tree was constructed using the neighbor-joining method (MEGA 6.0). The red blot indicates the position of TaWAK7D.

Using RT-PCR and 3′-RACE methods, a full-length cDNA (2373-bp) of the *TaWAK7D* gene was obtained from the resistant wheat cv. CI12633 and has been deposited in GenBank (accession number MW816106). It includes an open reading frame (ORF) with 2178-bp length and 3′-untranslated region (3′-UTR) with 195-bp. Comparision showed that the genomic sequence contains three exons and two introns (Figure 2A). The predicted TaWAK7D protein is consisted of 725 amino acid (aa) residues with a predicted Mw of 79.56 kDa and theoretical pI of 5.72. As shown in Figure 2B, the TaWAK7D protein contains an extracellular GUB domain (no. 3–62 amino acids, aa), an EGF domain (no. 226–274 aa), an EGF-calcium binding domain (EGF-CA, no. 275–319 aa), a transmembrane region (329–351) and an intracellular conserved Ser/Thr kinase domain (no. 399–669 aa). 

TaWAK7D protein and 15 WAK proteins from various plant species were applied to construct the phylogenetic tree. The results indicated that these 16 WAK proteins are grouped into two categories (Figure 2C). All monocot WAKs were clustered onto the same branch, including *Triticum aestivum* TaWAK7D, TaWAK3, and TaStb6, ***Z****ea mays* ZmqHSR1 and ZmHtn1; *Oryza sativa* OsXa4*; Hordeum vulgare* HvWAK3; *Aegilops tauschii subsp*. *tauschii* AeWAK2 and AeWAK3. All WAKs of dicots plants were clustered onto another branch, including *Arabidopsis thaliana* AtWAK3; *Arabidopsis halleri AhWAK5; Arabis alpina* AaWAK4; *Brassica rapa* BrWAK2; *Solanum lycopersicum* SlWAK3; *Capsicum annuum* CaWAK1; and *Nicotiana attenuata* NaWAK2. It suggested that the WAKs differentiation might occur during monocot–dicot divergence. Interestingly, the TaWAK7D protein was closely related to *Aegilops tauschii* AeWAK3 with 99.59% identity, implying that the TaWAK7D might originate from the *Aegilops* *tauschii* AeWAK3 (Figure S1).

### 2.3. TaWAK7D Localizes at the Plasma Membrane

**Figure 3:**
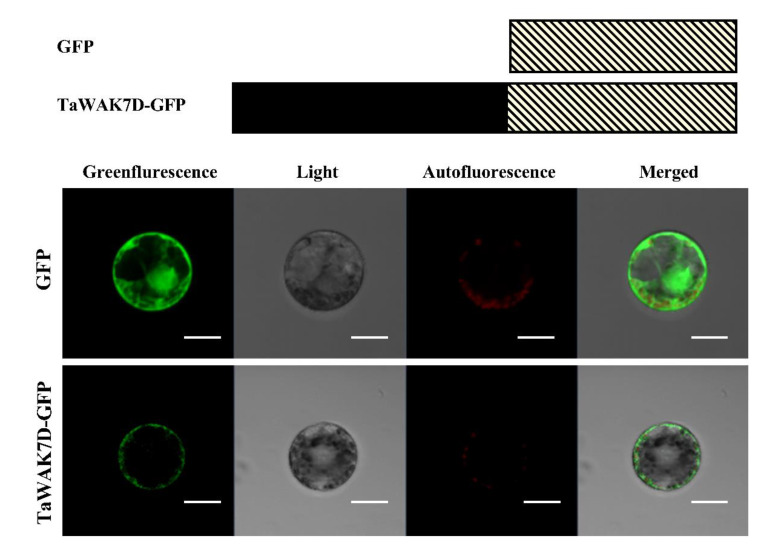
Subcellular localization of TaWAK7D in wheat protoplasts cells. The control GFP and fused TaWAK7D–GFP are transiently expressed in mesophyll protoplasts cells. The red colour was auto-fluorescence from wheat chloroplast. Scale bars = 20 μm (wheat protoplasts).

To investigate the subcellular localization of TaWAK7D in wheat plant cells, the pCaMV35S:TaWAK7D-GFP vector was constructed. The TaWAK7D-GFP fusion protein and GFP control protein were separately introduced into wheat mesophyll protoplasts and then transiently expressed (Figure 3). Confocal microscopic examination showed that the TaWAK7D-GFP protein was distributed at the plasma membrane in wheat, and the control GFP protein diffused both in the nucleus and cytoplasm (Figure 3). These results indicated that TaWAK7D should localize at the plasma membrane. 

### 2.4. TaWAK7D Is Required for Wheat Resistance against R. cerealis

**Figure 4:**
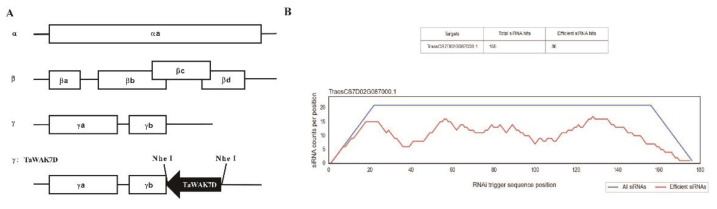
Schemata of recombinant BSMV:TaWAK7D construct and si-Fi software off-target prediction. (**A**) Schemata of recombinant BSMV:TaWAK7D construct. The orientation of the *TaWAK7D* insert is indicated by dark box. (**B**) SIFI software off-target prediction results.

**Figure 5:**
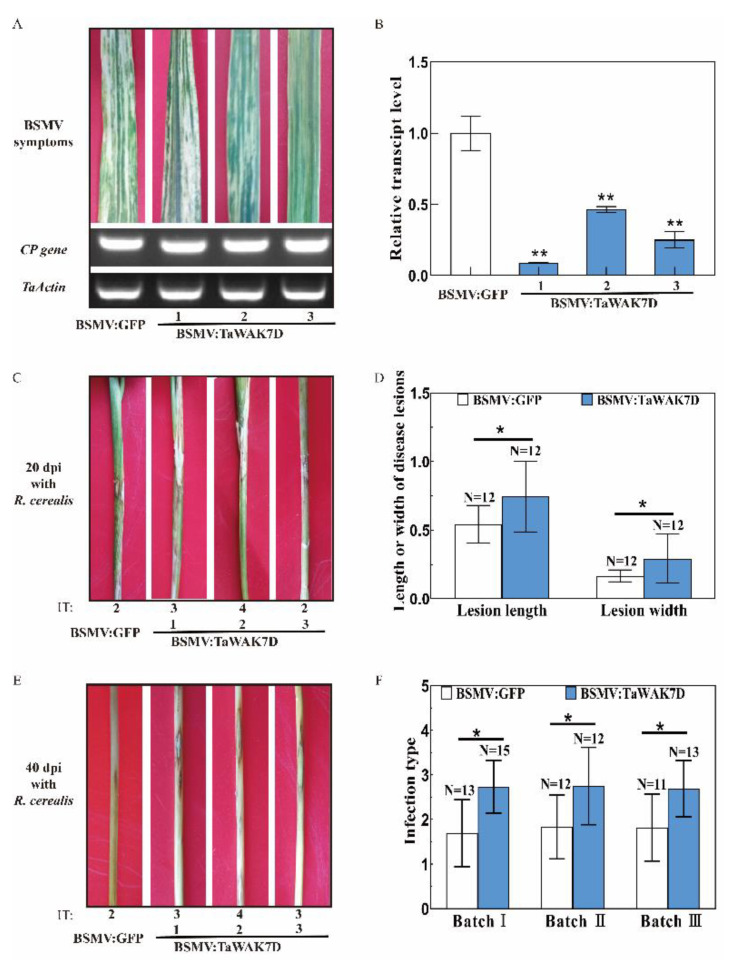
Silencing of *TaWAK7D* impairs resistance of the wheat cv. CI12633 to *R. cerealis*. (**A**) Typical symptom of BSMV in the fourth leaves of wheat plants infected by BSMV:GFP or BSMV:TaWAK7D at 15 dpi with BSMV and RT-PCR analysis of the transcription of the BSMV *CP* gene. (**B**) qRT-PCR analysis of the relative transcript level of *TaWAK7D* in BSMV:GFP or BSMV:TaWAK7D wheat plants. The transcript level of *TaWAK7D* in BSMV:GFP-infected wheat CI12633 plants was set to 1. Significant differences were determined based on three technical repeats (*t*-test: * *p*<0.01) (**C**) Sharp eyespot symptoms of BSMV:TaWAK7D- and BSMV:GFP-inoculated CI12633 plants at 20 dpi with *R. cerealis*. Bar represents 1 cm. (**D**) Disease lesion size in *TaWAK7D*-silencing and BSMV GFP control CI12633 plants at 40 dpi with *R. cerealis*. Significant differences were determined based on 12 independent biological replications. Bars indicate standard error of the mean. (**E**) Sharp eyespot symptoms of the BSMV:GFP and BSMV:TaWAK7D-inoculated CI12633 plants at 40 dpi with *R. cerealis*. IT indicates sharp eyespot infection type of each wheat plant. (**F**) Mean infection types of CI12633 plants infected by BSMV:GFP or BSMV:TaWAK7D in three batches. Significant differences were determined based on 11–15 independent biological replications (Student’s *t*-test: * *p* < 0.05). Bars indicate standard error of the mean.

**Figure 6:**
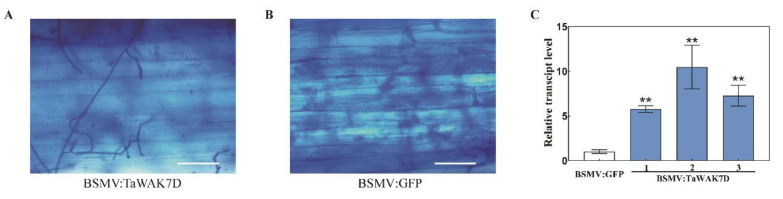
Trypan blue staining and *RcActin* transcript level in BSMV:TaWAK7D- and BSMV:GFP-infected wheat CI12633 plants. Trypan blue staining for the detection of the *R. cerealis* hyphae on the base leaf sheath of the BSMV:TaWAK7D (**A**) and BSMV:GFP (**B**) inoculated CI12633 plants at 4 dpi with *R. cerealis* Rc207. Bar = 20 μm. (**C**) qRT-PCR analysis of *RcActin* gene in stems of BSMV:TaWAK7D- and BSMV:GFP-infected wheat CI12633 plants at 10 dpi with *R. cerealis*. *RcActin* transcription represents the relative biomass of *R. cerealis.* Significant differences were determined based on three technical repeats (Student’s *t*-test: ** *p* < 0.01). The expression level of *RcActin* in BSMV:GFP-infected wheat CI12633 plants was set to 1.

The barley stripe mosaic virus (BSMV)-mediated VIGS approach was used to investigate the defense role of *TaWAK7D* against *R. cerealis* infection. In this study, the si-Fi software was used to predict and design optimization of RNAi constructs [24], then a 175 bp fragment of *TaWAK7D* 3′–UTR was subcloned in antisense orientation into the RNA γ of BSMV, to form a BSMV:TaWAK7D recombinant vector (Figure 4). At 15 days post transfection of BSMV-derived RNAs into leaves of the resistant wheat cv. CI12633, infection symptoms of BSMV appeared on newly emerged leaves and the transcript of BSMV coat protein gene (*cp*) could be detected (Figure 5A), indicating that BSMV infected these wheat plants. Meantime, qRT-PCR analyses showed that the transcript level of *TaWAK7D* was significantly lower in BSMV:*TaWAK7D*-infected CI12633 plants compared to BSMV:GFP-infected CI12633 plants (Figure 5B), indicating that *TaWAK7D* was successfully silenced in BSMV:TaWAK7D-infected CI12633 plants, hereafer named *TaWAK7D*-silenced CI12633 plants. Subsequently, these plants were inoculated with *R. cerealis* Rc207. At 4 dpi with *R. cerealis*, the microscope observation showed that the hyphae of *R. cerealis* were more on the infected sheaths of *TaWAK7D*-silenced CI12633 plants than those of the BSMV:GFP-infected CI12633 plants (Figure 6A,B). Accordingly, the relative biomass of the fungus, represented by *RcActin* transcript level, was higher (6-fold) in *TaWAK7D*-silenced CI12633 plants than in BSMV:GFP-infected CI12633 plants (Figure 6C). After 20 dpi with *R. cerealis*, typical symptoms of sharp eyespot appeared and the greater necrosis sizes of the disease on the stems of *TaWAK7D*-silenced CI12633 plants, compared to BSMV:GFP-treated CI12633 plants (Figure 5C). Moreover, at 40 dpi with *R. cerealis*, larger necrotic areas and significantly higher disease severity of sharp eyespot appeared on the stems of *TaWAK7D*-silenced CI12633 plants compared to BSMV:GFP-infected CI12633 plants, (Figure 5E). The average necrotic length and width of *TaWAK7D*-silenced CI12633 plants were 0.74 and 0.29 cm, whereas the BSMV:GFP-infected CI12633 plants were 0.54 and 0.16 cm (Figure 5D). In three batches of VIGS experiments, the results of disease scoring showed that the average ITs of *TaWAK7D*-silenced CI12633 plants were 2.69 to 2.75, respectively, whereas the average ITs of BSMV:GFP-treated CI12633 plants were 1.69 to 1.81, respectively (Figure 5F). These results clearly indicated that silencing of *TaWAK7D* compromised resistance of the host ci12633 to sharp eyespot, and suggested that *TaWAK7D* expression was required for wheat resistance to *R. cerealis* infection. 

### 2.5. TaWAK7D Activates the Expression of Defense Genes

**Figure 7:**
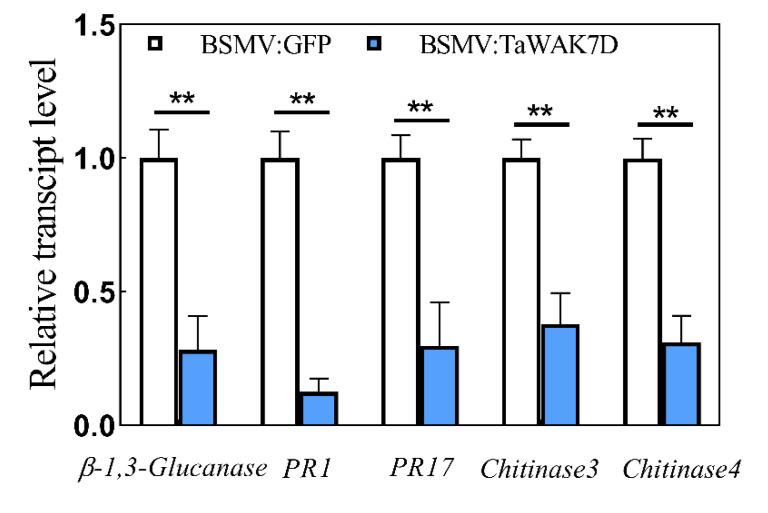
Transcript profiles of *TaWAK7D* and pathogenesis-related genes in BSMV:GFP- and BSMV:TaWAK7D-infected wheat plants after infection by pathogens. Relative transcript abundances of *β-1,3-Glucanase*, *PR1*, *PR17 Chitinase3* and *Chitinase4* in BSMV:TaWAK7D-infected CI12633 plants were quantified relative to those in BSMV:GFP-infected control plants after *R. cerealis* inoculation for 10 days. Statistically significant differences between BSMV:TaWAK7D-infected and BSMV:GFP-infected wheat plants were determined based on three biological replications (Student’s *t*-test: ** *p*< 0.01). Bars indicate the standard error of the mean. *TaActin* was used as an internal control.

Some pathogenesis-related genes, important defense genes, have been shown to participate positively in the wheat resistance response to *R. cerealis* [25]. To investigate if *TaWAK7D* is required for the expression of pathogenesis-related genes in wheat defense response to *R. cerealis*, qRT-PCR was used to examine the transcript levels of pathogenesis- related genes in *TaWAK7D*-silenced wheat and the BSMV:GFP-infected control plants inoculated with *R. cerealis* for 10 days. The tested genes include *β-1,3-Glucanase*, *Chitinase3*, *Chitinase4*, *PR1* and *PR17*. The analyses showed that the transcript levels of *β-1, 3-Glucanase*, *Chitinase3*, *Chitinase4*, *PR1* and *PR17* significantly decreased in *TaWAK7D*-silenced CI12633 plants relative to the BSMV:GFP-infected CI12633 plants (Figure 7). These results suggested that *TaWAK7D* expression might be required for the expression of these five *PR* genes in the wheat response to *R. cerealis*.

### 2.6. TaWAK7D May Contribute to Pectin- and Chitin-Induced Immune Responses

**Figure 8:**
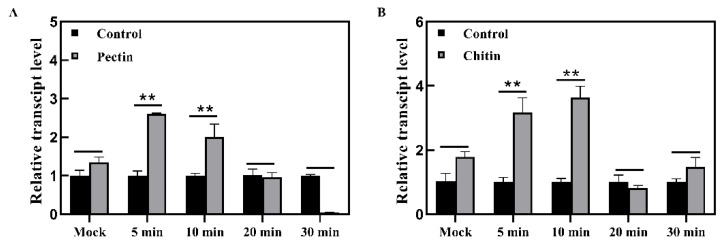
Transcript profiles of *TaWAK7D* responding to exogenous pectin and chitin treatments. (**A**) Transcript profiles of *TaWAK7D* in wheat cv. CI12633 leaves treated by 100 μg/mL exogenous pectin. (**B**) Transcript profiles of *TaWAK7D* in leaves of wheat cv. CI12633 after exogenous application of 100 μg/mL chitin. The transcript level of *TaWAK7D* in mock-treated wheat plants is set to 1. Statistically significant differences are analyzed based on three biological replications (Student’s *t*-test: ** *p* < 0.01). Error bars indicate the SE.

Chitin and pectin are conserved components of fungal cell wall, which can trigger plant immune responses [11,26]. To investigate how *TaWAK7D* responds to exogenous pectin and chitin stimuli, we analyzed the transcriptional profiles of *TaWAK7D* in wheat cv. CI12633 treated with 100 μg/mL pectin or chitin, as well as mock treatments for mock, 5, 10, 20 and 30 min. *TaWAK7D* transcript levels were significantly elevated by pectin and chitin treatments, compared with the mock treatment (Figure 8A,B). These results suggested that *TaWAK7D* might be involved in pectin- and chitin-induced immune responses in wheat.

## 3. Disscusion

In this study, we identified a novel wheat WAK-encoding gene *TaWAK7D* and analyzed its role in defense responses to *R. cerealis. TaWAK7D* is located on wheat chromosome 7D. The transcript level of *TaWAK7D* was not only significantly induced after infection of *R. cerealis*, but also was higher in sharp eyespot-resistant wheat cultivars than in susceptible wheat cultivars. Importantly, silencing of *TaWAK7D* impaired resistance of wheat to sharp eyespot caused by *R. cerealis* infection. The results suggest that *TaWAK7D* expression is required for resistance to sharp eyespot in wheat. In contrast, although another WAK gene *TaWAK5* transcript abundance was induced in a stronger extent in resistant wheat genotypes than in susceptible ones after *R. cerealis* infection, silencing of *TaWAK5* did not change obviously the resistance in wheat, presumably due to the existence of functional redundancy among the genes in this gene family [23]. Previous studies showed that *WAK* genes are involved in disease resistance in other plant species. In cotton, *GhWAK7A* silencing increases cotton susceptibility to Verticillium and Fusarium wilts [27]. In rice, loss-of-function mutants in *OsWAK91* reduce resistance ability to *M. oryzae*, and overexpressing *OsWAK91* plants enhance the resistance ability, while the mutant in the *OsWAK112d* and overexpression of the *OsWAK112d* led to increased resistance and susceptibility, respectively [17]. In wheat, mutation or siliencing of *Stb6* (*TaWAKL4* gene) both compromised resistance to STB disease, while the *Stb6-*overexpressing transgene plants became more resistant than the WT plants [21]. This study extends the current knowledge of plant *WAKs* in plant innate immune responses to necrotrophic pathogens. 

The TaWAK7D protein sequence contains all typical WAK domains: a galacturonan-binding GUB domain, an EGF domain, an EGF-calcium binding (EGF-CA) domain, a transmembrane region TM and a Ser/Thr kinase domain [28]. Previous papers reported that a non-RD kinase domain typically was found in plant innate immune receptors, and non-RD-type proteins reported were responsible for triggering a cascade of intracellular events during defense responses [29]. For instance, the ZmWAK-RLK1 encoded by *Htn1* and *ZmqHSR-*encoding ZmWAK as well as the rice Xa4 protein sequences all contain a non-RD kinase domain [13,15,20]. Sequence analysis and phylogenetic analysis revealed that TaWAK7D is a non-RD-type WAK protein in wheat. Additionally, TaWAK6 is a non-RD-type WAK, and its overexpression confers wheat resistance to leaf rust, similar to adult plant resistance [22]. Mostly recently, *TaStb6,* which confers resistance against infection of *Zymoseptoria tritici,* encodes a RD-type WAK [21]. Additionally, certain *Arabidopsis* defense-associated LRR-RLKs, such as brassinosteroid insensitive 1-associated receptor kinase [30], Flg22-induced receptor-like kinase 1 (FRK1) [31], the PEPtide 1 and PEPtide 2 receptors [32] are all RD kinases. Interestingly, the TaWAK7D protein was localized to the plasma membrane in wheat mesophyll protoplasts. Some disease-resistant WAKs have been reported to localize at the plasma membrane. For example, the *Xanthomonas citri* subsp. *citri* (Xcc) resistance protein CsWAKL08 [33]*,* the *Magnaporthe oryzae* resistance protein OsWAK1 [16] and the maize ZmHtn1, which confers quantitative resistance to *Exserohilum turcicum* [15], were all reported to be localized in the plasma membrane. These findings suggest that the plasma membrane distribution of these RLKs might meet their immune receptor roles.

The heightened expression of pathogenesis-related genes positively contributes to plant defense against pathogens. Previous studies indicated that several PR-encoding genes, such as *β-1,3-Glucanases*, *Chitinases, PR1* and *PR17*, contributed to the resistance of wheat to sharp eyespot caused by *R. cerealis* [34,35]. To explore the molecular mechanism underlying the defensive role of *TaWAK7D*, we investigated the transcripts of a subset of pathogenesis-related genes in *TaWAK7D*-silenced wheat and the control plants. The results showed that after *R. cerealis* inoculation, the transcript levels of *β-1,3-Glucanase*, *Chitinase3*, *Chitinase4, PR1* and *PR17* were down-regulated in more susceptible *TaWAK7D*-silenced CI12633 plants than in the control plants. The data suggest that TaWAK7D might indirectly activate the expression of the above defense molecules in wheat resistance responses against *R. cerealis*. Previous studies showed that overexpressing *OsWAK25* enhanced resistance to the hemibiotrophic pathogens *X. oryzae* and *M. oryzae* through activating expression of *PR10* and *PBZ1* in rice [19], and the maize smut resistance gene *ZmqHSR1* (*ZmWAK*) elevated the expression of *ZmPR-1* and *ZmPR5* [13]. These findings suggest that these WAK proteins elevate the expression of certain PR genes, resulting in enhanced resistance. In a previous study, WAKs have been reported to be associated with the perception of cell wall components in *Arabidopisis* [10]. In this study, we found that *TaWAK7D* transcript levels were significantly elevated after pectin and chitin treatments, suggesting that *TaWAK7D* may involved in pectin- and chitin-induced immune responses.

## 4. Conclusions

We identified a novel wheat wall-associated kinase gene *TaWAK7D* in the defense responses to *R. cerealis* infection. The active *TaWAK7D* is required for wheat resistance responses to *R. cerealis* and the expression of at least five pathogenesis-related genes, including *β-1,3-glucanase*, *Chitinase3*, *Chitinase4, PR1* and *PR17*. Thus, the expressed *TaWAK7D* positively regulates the innate immune responses to *R. cerealis* through activating the expression of these pathogenesis-related genes in wheat. *TaWAK7D* is a candidate gene in improving wheat resistance to *R. cerealis* infection. 

## 5. Materials and Methods

### 5.1. Plant and Fungal Materials, Primers and Treatments

Four wheat (*Triticum aestivum*) cultivars—CI12633, Shanhongmai, Wenmai 6 and Yangmai 9—exhibiting different levels of resistance and susceptibility to sharp eyespot [8,35], were used to investigate *TaWAK7D* transcript profiles. The resistant wheat cv. CI12633 was used in a virus-inducing gene silencing (VIGS) experiment. *R. cerealis* strain Rc207, which is highly virulent in north China, was provided by Prof. Jinfeng Yu, Shandong Agricultural University, China. 

All wheat plants were grown in a greenhouse under 23 °C/14 h light and 15 °C/10 h dark. At the tillering stage, the stem base of each plant was inoculated with toothpick fragments harboring well-developed mycelia of *R. cerealis*. The inoculated sites were covered with wet cotton to increase the humidity, which promotes *R. cerealis* infection. Inoculated plants were grown at 90% relative humidity for 4 days. At 4- and 10-dpi with *R. cerealis* Rc207 or mock inoculation, nine plants derived from three resistant RILs and three susceptible RILs were sampled for deep RNA-sequencing and comparative transcriptomic analysis. The sequences of all primers in this study are listed in Table S1. 

### 5.2. RNA Extraction and qRT-PCR

Trizol reagent (Invitrogen, Life Technologies, Carlsbad, CA, USA) was used to extract wheat tissues’ total RNA from different wheat cultivars. Then, the RNA was purified and reverse-transcribed into cDNA using the FastQuant RT Kit (Tiangen, Beijing, China), which was used in RT-PCR or real-time quantitative PCR (qRT-PCR). In RT-PCR, the transcription level of a BSMV coat protein (CP) gene was measured to check whether the BSMV was successfully infected into the wheat plants. In qRT-PCR, specific primers were used to measure the transcription level of the *TaWAK7D*, *β-1,3-Glucanase*, *Chitinase3*, *Chitinase4*, *PR1* and *PR17* in wheat plants. qRT-PCR was performed on an ABI7500 instrument (Applied Biosystems, Waltham, MA, USA) with a SYBR Premix ExTaq kit (Takara Bio Inc., Otsu, Japan).

Reactions were programmed with the following thermal cycling profile: 95 °C for 3 min, followed by 40 cycles of 95 °C for 5 s, 58 °C for 10 s and 72 °C for 32 s. The PCR products were loaded onto 1.5% agarose gels and visualized under UV after staining with ethidium bromide. Each experiment was replicated three biological times. The relative expression of target genes was calculated using the 2^−ΔΔCT^ method, with the wheat actin gene *TaActin* used as internal reference gene [36]. 

### 5.3. Cloning and Sequence Analyses of TaWAK7D

The full-length open reading frame (ORF) sequence of WAK7D was amplified with specific primers TaWAK7DF/R from cDNA of CI12633 plants. The 3′–UTR of TaWAK7D was amplified by RACE (rapid-amplification of cDNA ends) method. The PCR products were cloned into pMD18-T vector (Takara Bio Inc., Otsu, Japan) and then sequenced. The predicted protein sequence was analyzed with the Compute pI/Mw tool (http://web.expasy.org/compute_pi/, accessed on 9 July 2020) to determine the theoretical pI (isoelectric point) and Mw (molecular weight), interPro-Scan (http://www.ebi.ac.uk/interpro/, accessed on 9 July 2020) to identify domains and Smart software (http://smart.emblheidelberg.de/, accessed on 9 July 2020) to predict conserved motifs. A phylogenetic tree was constructed using a neighbor-joining method implemented in MEGA 6.0 software (https://www.megasoftware. net/, accessed on 9 July 2020) after alignment with other WAK protein sequences using ClustalW software (https://www.genome.jp/tools-bin/clustalw, accessed on 9 July 2020). 

### 5.4. Subcellular Localization of TaWAK7D

The coding region of *TaWAK7D* lacking the stop codon was amplified using gene-specific primers TaWAK7D-GFP-F/TaWAK7D-GFP-R. The amplified fragment was digested with restriction enzyme *Bam*H I, and subcloned in-frame into the 5′-terminus of the GFP (green fluorescent protein) coding region in the pCaMV35S:GFP vector, resulting in the TaWAK7D-GFP fusion construct pCaMV35S:TaWAK7D-GFP. The p35S:TaWAK7D-GFP fusion construct or p35S:GFP control construct was separately individually introduced into wheat mesophyll protoplasts [37,38]. After incubation at 25 °C for 16 h, GFP signals were observed, and photographed using a confocal laser scanning microscope [39] (Zeiss LSM 700, Germany) with a Fluor×10/0.50 M27 objective lens and SP640 filter. 

### 5.5. Virus-Induced Gene Silencing (VIGS) Assay for TaWAK7D

Barley stripe mosaic virus (BSMV)-mediated VIGS has been successfully utilized to study gene function in barley and wheat [40]. In this study, a175bp fragment of *TaWAK7D* 3′–UTR was subcloned in antisense orientation into the *Nhe* I restriction site of the RNA γ of BSMV, to form a BSMV:TaWAK7D recombinant vector (Figure 4). Then, the tripartite cDNA chains of BSMV:TaWAK7D or the control BSMV:GFP virus genomes were separately transcribed into RNAs, mixed and used to infect CI12633 seedlings at the three-leaf stage. At 15 dpi, the fourth leaves of the inoculated seedlings were collected to monitor BSMV infection, to analyze the transcription of BSMV *CP* gene and to evalued the relative transcript change of *TaWAK7D* in BSMV:GFP or BSMV:TaWAK7D wheat plants.

### 5.6. Functional Assay of TaWAK7D-Mediated Defense against R. cerealis in Wheat

Functional assays of *TaWAK7D*-mediated defense against *R. cerealis* in wheat were determined based on three independent biological repetitions, 13 and 15, 12 and 12 and 11 and 13 of control and *TaWAK7D*-silenced wheat plants, respectively, were inoculated with small toothpicks (about 3 cm) harboring the well-developed mycelia of *R. cerealis.* Seven dpi with *R. cerealis*, leaf sheathes of BSMV:TaWAK7D and BSMV:GFP-infected CI12633 plants were staining in the Trypan blue solution for 5 min, and then checked whether the hyphaes of *R. cerealis* infected the wheat plant cells under a microscope. At 20 and 40 dpi, sharp eyespot infection types (ITs) of wheat plants were pictured and scored as previously described [4,41]. Average lesion length and lesion width was used to represent a lesion size.

## Data Availability

All data supporting the findings of this study are available within the paper and its supplementary materials published online.

## Supplementary Materials

The following are available online at https://www.mdpi.com/article/10.3390/ijms22115629/s1, Figure S1: Amino acid sequence alignment of TaWAK7D and AeWAK3, Table S1: Primers and their sequences used in this study.
