# Supplementary material for: The Wall-Associated Receptor-Like Kinase TaWAK7D Is Required for Defense Responses to Rhizoctonia cerealis in Wheat
Source: Int J Mol Sci. 2021 May 26;22(11):5629. doi: 10.3390/ijms22115629 (PMC8199179; doi:10.3390/ijms22115629)
Supplement: Supplementary file 1 [file ijms-22-05629-s001.zip › WAK7DSupplMaterials.pdf]

Supplementary Materials:

Figure S1 Multiple amino acid sequences alignment of TaWAK7D and AeWAK3.

Table S1 Primers and their sequences used in this study.

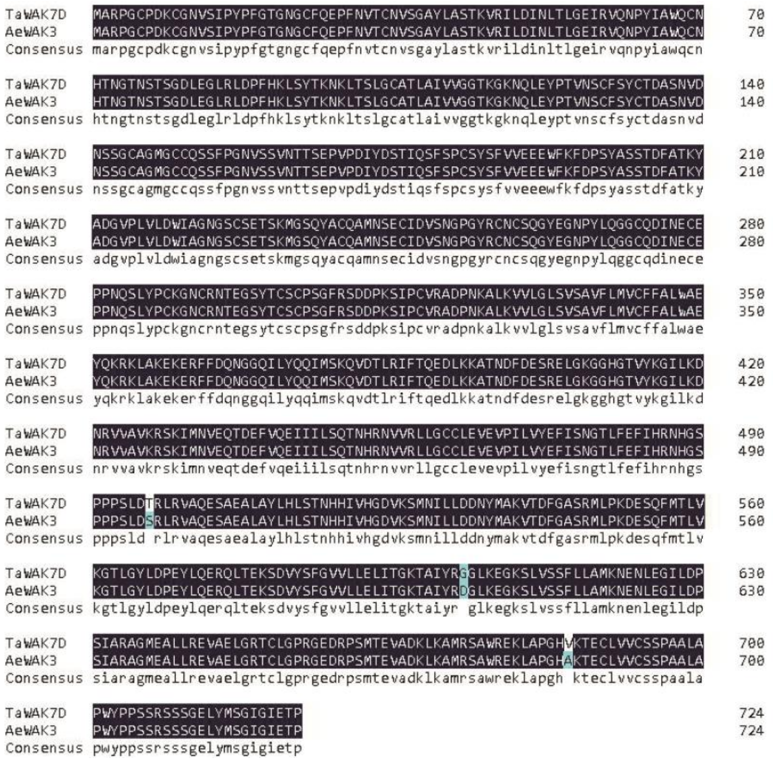

Figure S1 Multiple amino acid sequences alignment of TaWAK7D and AeWAK3.

Table S1 Primers and their sequences in this study.

Primer name	Sequence (5´–3´)	Use
7D87-F1	5'-TCACAGAGAAGAGCGACG-3'	PCR for cDNA amplification
7D87-F2	5'-CCTCGTGTCATCCTTCCT-3'	PCR for cDNA amplification
oligdT	5′- TACCGTCGTTCCACTAGTGATTTTTTTTTTT-3′	PCR for cDNA3' RACE amplification
7D8700-VIGSF4	5'-TAC<u>GCTAGC</u>CATTCGAGCCTTCTACCAA-3'	VIGS
7D8700-VIGSR4	5'-GAC<u>GCTAGC</u>GTGTGCGTGTGTTTCTTC-3'	VIGS
GFP-7D087-inF	5'-TATCTCTAGAGGATCCATGGCACGGCCCGGC TGCCCAGACAAGTGCGGTAAC-3'	subcellular localization
GFP-7D087-inR1	5'-TGCTCACCATGGATCCTCTGGGTGTCTCTATG CC-3'	subcellular localization
7D087-RTF1	GGCACTGTCTACAAGGGCAT	RT-qPCR
7D087-RTR1	GGTCTGCTCCACGTTCATGA	RT-qPCR
qTaActinF	GGAATCCATGAGACCACCTAC	RT-qPCR
qTaActinR	GACCCAGACAACTCGCAAC	RT-qPCR
BSMV-CPF	5'-TGACTGCTAAGGGTGGAGGA-3'	RT-PCR
BSMV-CPR	5'-CGGTTGAACATCACGAAGAGT-3'	RT-PCR
RcActin-F	5'-GCATCCACGAGACCACTTAC-3'	RT-qPCR
RcActin-R	5'-GCGTCCCGCTGCTCAAGAT-3'	RT-qPCR
β-1,3-Glucanase-F	5'-GCGTGAAGGTGGTGATTT-3'	RT-qPCR
β-1,3-Glucanase-R	5'-GTGCCCGTTACACTTGGAT-3'	RT-qPCR
Chitinase3-F	5'-CCCACCCTAACCTGAGCATC-3'	RT-qPCR
Chitinase3-R	5'-ACTGGTTGATCATGGCGGAG-3'	RT-qPCR
Chitinase4-F	5'-GAAGTCCCCCATGGCGATC-3'	RT-qPCR
Chitinase4-R	5'-GGTCCCGCAATAACCGTACT-3'	RT-qPCR
PR1-F	5'-AAACAGCAGCAACCCAAGAA-3'	RT-qPCR
PR1-R	5'-GGGTCCAGTAGCACCGATTTA-3'	RT-qPCR
PR17-F	5'-CGAAAAGGGAGCGGTGGATT-3'	RT-qPCR
PR17-R	5'-CGGGAAATGGGGCACAAC-3'	RT-qPCR
